# Syringocystadenoma Papilliferum: A Challenging Clinical Diagnosis in a Patient With a Persistent Scalp Lesion After Trauma

**DOI:** 10.7759/cureus.68960

**Published:** 2024-09-08

**Authors:** Fawaz H Aljehani, Mehad F Almoqati, Nawras A Alyamani, Walaa A Ahmed, Tala M Roblah, Lama Z Allehaibi

**Affiliations:** 1 Dermatology, King Abdul Aziz Hospital, Makkah, SAU; 2 Medicine and Surgery, Taif University, Taif, SAU; 3 Medicine and Surgery, Umm AlQura University, Makkah, SAU; 4 Medicine and Surgery, Umm AlQura university, Makkah, SAU; 5 Medicine and Surgery, King Abdulaziz University, Jeddah, SAU; 6 Medicine, Umm AlQura University, Makkah, SAU

**Keywords:** adnexal tumor, benign tumor, nevus sebaceous, scalp lesion, syringocystadenoma papilliferum

## Abstract

Syringocystadenoma papilliferum is a rare, hamartomatous benign tumor originating from either the eccrine or apocrine sweat glands. We report a case of a 30-year-old female who presented with a 10-year history of an asymptomatic, slow-growing scalp lesion following head trauma. A scalp examination revealed a single, rounded 3 mm fleshy erythematous nodule with a central crust in the right parietal area. a biopsy revealed downward papillomatous extensions in the epidermis and multiple epithelial sheets with dilated ducts lined by columnar cells in the dermis. A diagnosis of syringocystadenoma papilliferum was confirmed based on these clinicopathological findings. The patient was reassured and underwent complete surgical excision of the lesion.

## Introduction

Syringocystadenoma papilliferum (SCAP) is a rare benign hamartomatous adnexal tumor originating from either the eccrine or apocrine sweat glands [1,2]. It is often associated with other benign adnexal lesions such as sebaceous nevus, apocrine nevus, tubular apocrine adenoma, apocrine hidrocystoma, apocrine cystadenoma, and clear cell syringoma [3]. Although SCAP is benign, it has the potential to progress to basal cell carcinoma (BCC), metastatic adenocarcinoma, and ductal carcinoma [3]. The most common associated malignancy is BCC, which has been reported in 10% of the cases [4].

Mostly it is a childhood tumor, about 50% of affected individuals have the tumor since birth or early childhood, and another 15% to 30% develop it before puberty [1,3]. A majority of SCAPs arise on the head and neck. Other unusual locations include the buttock, vulva and scrotum, pinna, eyelid, outer ear canal, surgical scar, scalp, nipple, thigh, axilla, back, and right lower abdomen [3,5]. Macroscopically, mature SCAP lesions appear as clusters of generally pinkish-brown nodules, ranging in size from 2-10 mm in diameter, with an occasional central opening [6]. The typical microscopic appearance consists of ducts connecting to the surface, lined by two epithelial cell layers, and containing papillary processes [5].

The tumor has a widely variable clinical appearance. The type of plaque that appears as a hairless area on the scalp is frequently linked to a sebaceous nevus of Jadassohn. linear lesions are more commonly seen in the face and neck, while single nodular lesions typically appear on the trunk [1].

In this paper, we are presenting a case that was clinically diagnosed as syringocystadenoma papilliferum with histopathological confirmation.

## Case presentation

**Figure 1:**
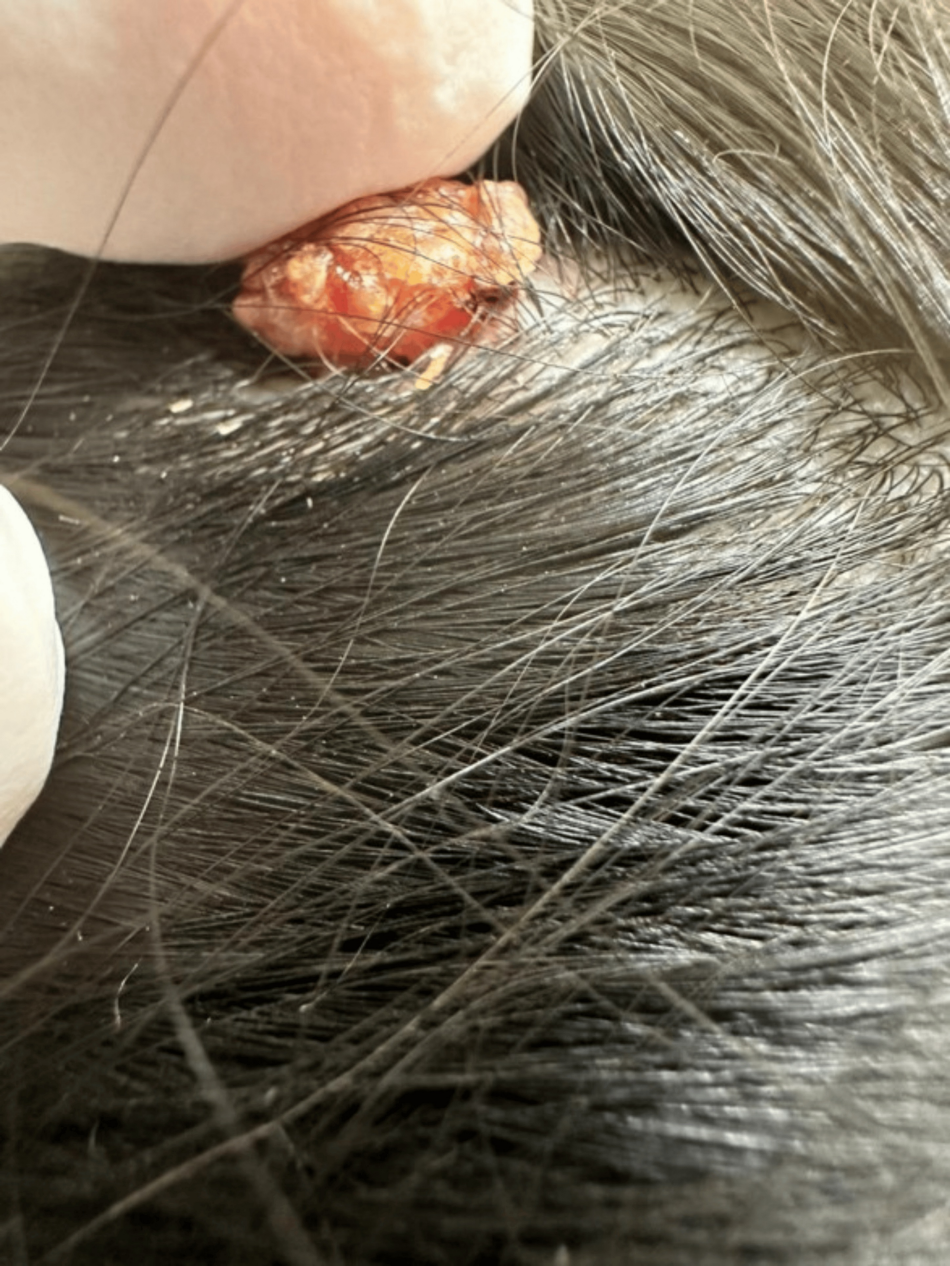
The image shows a single, fleshy erythematous nodule with central crustation on the right parietal region of the scalp, measuring 3 mm in size.

**Figure 2:**
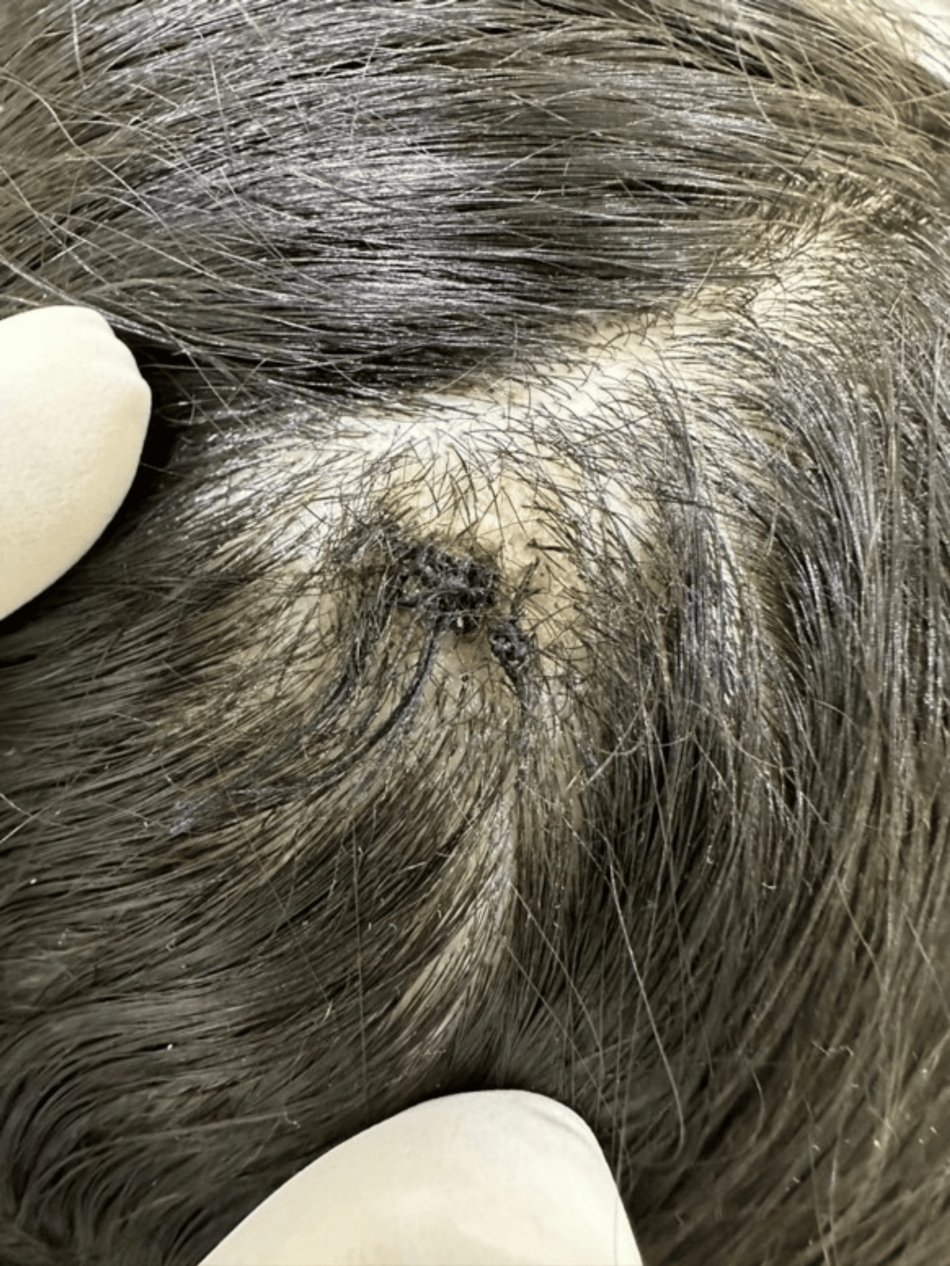
A 6mm punch biopsy with a free margin was performed under local anesthesia, excising the lesion completely.

A 30-year-old female presented with an asymptomatic solitary erythematous nodule measuring 3 mm on the scalp and persisting for 20 years. The patient first noticed the lesion after a traumatic head injury that happened when she was nine years old. The nodule remained constant in size until April 2024, but it began to enlarge thereafter, prompting the patient to seek medical attention. However, there were no changes in the appearance of the nodules or characteristics. The nodule was soft in consistency, well-demarcated, and had a smooth surface. There were no associated skin lesions or hair growth on the lesion. Nevertheless, the patient reported that whenever she combed her hair the nodule oozed clear fluid. Additionally, there was no family history of similar complaints. On scalp examination, a single fleshy erythematous nodule measuring 3 mm with central crustation was noted on the parietal region of the scalp (Figure 1). The rest of the scalp appeared normal, with no evidence of local lymphadenopathy. Differential diagnoses include syringocystadenoma papilliferum, papillary eccrine adenomas, hidradenoma papilliferum, warty dyskeratoma, tubular apocrine adenoma, and inverted follicular keratosis. A 6 mm punch biopsy with a free margin was performed under local anesthesia, excising the lesion completely (Figure 2).

**Figure 3:**
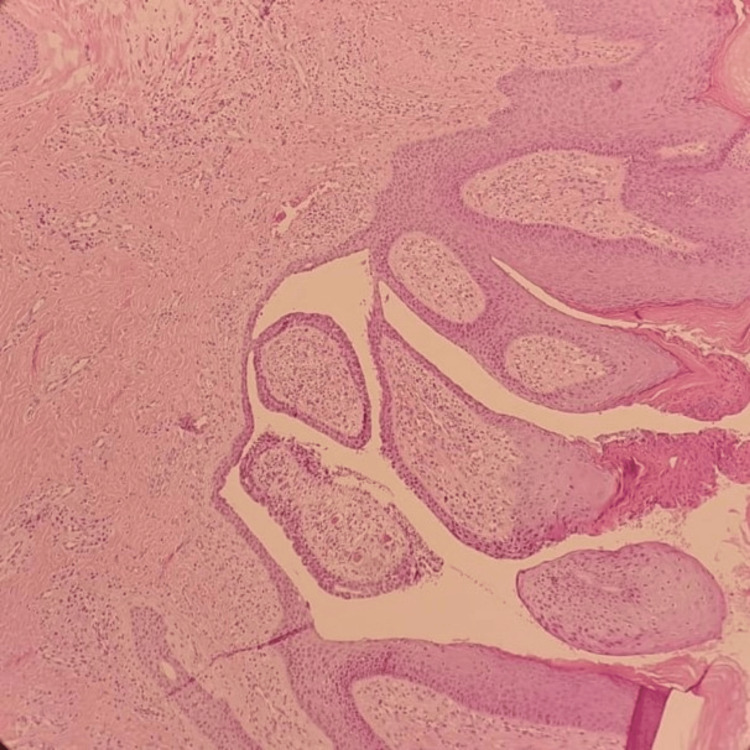
Histopathology of the erythematous nodule A 6 mm punch biopsy with a free margin revealed exophytic epidermal surface with multiple dilated glandular channels that opens from the skin surface and branches down into the underlying dermis. Papillary islandswith central fibrovascular cores float within the dilated channels and lined by double layer of bland cuboidal to columnar sweat duct type epithelium. The stroma of the tumor consists of an inflammatory infiltrate of plasma cells and lymphocyte.

The histopathological examination revealed an exophytic epidermal surface with multiple dilated glandular channels that open from the skin surface and branch down into the underlying dermis. Papillary islands with central fibrovascular cores float within the dilated channels and are lined by a double layer of bland cuboidal to columnar sweat duct-type epithelium. The stroma of the tumor consists of an inflammatory infiltrate of plasma cells and lymphocytes (Figure 3). Thus, based on histopathological findings, the patient was diagnosed with syringocystadenoma papilliferum.

## Discussion

SCAP is a rare, benign tumor that originates from the adnexal structures of the skin. The pathogenesis of SCAP is still not well understood, either sporadic or secondary neoplasms in sebaceous nevi [7]. SCAP is classified into three different types: solitary nodular, linear, and plaque. The solitary type presents as pedunculated nodules ranging from 5 to 10 mm in size and manifests near the proximal joints of the upper extremities [7,8]. While the linear type appears as multiple, small, erythematous papules in a linear pattern ranging from 1-10 mm in size. The plaque type is characterized by a hairless area with nodular or crusted plaques, as seen in our patient [7].

The exact etiology of SCAP remains unclear. However, there have been associations with certain genetic mutations as the activation of the RAS/RAF pathway is a consistent feature of SCAP [8]. Moreover, the involvement of HAS and BRAF V600 mutations contributes significantly to the tumorigenesis of sporadic SCAP [9]. HPV 6/11 infection has been linked to the verrucous SCAP on the buttock as reported by Skelton et al. [10]. In a different study, Konstantinova et al. observed features indicative of HPV infection, such as wart-like acanthosis and papillomatosis, in three out of 16 cases (18.8%) [9]. It is noteworthy that our patient had head trauma from a falling wooden plank before the tumor appeared, which may be an incidental finding because it is not previously documented in the literature. The benign presentation of SCAP typically manifests as a slowly enlarging, fleshy plaque that evolves over several years as seen in our patient [8]. In contrast, the malignant form of syringocystadenoma papilliferum is generally asymmetric, rapidly progressing, poorly delineated, and frequently invades the subcutaneous fat. Which was negative in our patient. Key factors that supported this benign diagnosis included histopathological findings which were consistent with benign syringocystadenoma papilliferum. However, excision removal is done to prevent conversion to the malignant type due to the frequent friction with daily hair combing [11].

Several differential diagnoses for SCAP were considered and meticulously ruled out through clinical evaluation and histopathological analysis. Papillary eccrine adenoma was excluded due to the absence of characteristic papillary structures and myoepithelial cells. Hidradenoma papilliferum was also considered but ruled out, given its typical anogenital location, whereas our case involved the scalp. Warty dyskeratoma was eliminated based on the lack of distinctive supra basilar clefting and acantholytic dyskeratosis. Tubular apocrine adenoma was dismissed owing to the absence of tubules lined by both luminal and myoepithelial cells. Lastly, inverted follicular keratosis was ruled out due to the absence of characteristic endophytic growth and keratin-filled cystic spaces [6]. While the observed histological features in our case were more consistent with SCAP.

"Histopathology is the gold standard for the diagnosis of syringocystadenoma papilliferum (SCAP). Microscopically, SCAP shows characteristic cystic invaginations originating from the epidermis, which is lined by a double layer of epithelial and myoepithelial cells [12]. In the epithelium lining these structures comprise two cell types: an outer layer of cuboidal or columnar cells and an inner layer of apocrine secretory cells [12]. Other characteristics include papillomatosis (finger-like projections), the presence of numerous plasma cells within the surrounding stroma, Irregular papillary architecture with cystic spaces or glands, and Loss of the typical double-layered epithelial lining in some areas. However, The malignant counterpart, syringocystadenocarcinoma papilliferum, exhibits characteristic histological findings such as asymmetric and poorly circumscribed growth, often extending deep into the subcutaneous fat, higher nuclear-to-cytoplasmic ratio, irregular nuclear features, coarse chromatin, and Increased mitotic activity compared to the benign syringocystadenoma papilliferum [12].

While the benign and malignant forms show overlapping microscopic features, the specific differences in architectural patterns, cytologic atypia, and mitotic activity help distinguish between these related adnexal neoplasms.

Additionally, immunohistochemical studies support the concept of SCAP as a hamartomatous tumor arising from pluripotent precursor cells. A study by Yamamoto et al. showed the luminal columnar cells were mostly positive for CK7 and CK19. These cells also showed heterogeneous expression of CK1\5\10\14, which was also observed in the sweat gland ductal/secretory cells. The basal cuboidal cells shared similarities with sweat gland basal/myoepithelial cells, expressing markers like CK1/5/10/14 and CK5/8. However, the basal tumor cells also showed heterogeneous expression of other markers like CK19, vimentin, and smooth muscle actin [13].

The mainstay of treatment for SCAP is complete surgical excision of the lesion [7]. Its importance is not only for a definitive diagnosis but also to prevent potential malignant transformation [12]. In cases where the lesion is located in cosmetically sensitive areas, alternative treatment options, such as CO2 laser may be considered [2]. Also, it is recommended that patients with SCAP be evaluated for associated malignancy and other potential malignant transformation [8]. As it is commonly associated with other malignant tumors such as basal cell carcinomas and verrucous carcinomas [1]. Additionally, it has been reported that up to 10% of SCAP cases develop basal cell carcinoma (BCC) [1].

## Conclusions

Syringocystadenoma papilliferum is a benign hamartomatous adnexal tumor that originates from either eccrine or apocrine sweat glands. Although diagnostically challenging, proper clinical examination and histopathological analysis are crucial for accurate diagnosis, as the tumor carries a risk of malignant transformation. Management often involves surgical excision, especially if the lesion becomes symptomatic or for aesthetic concerns. Our case was treated by surgical excision, with no recurrence until the time of the report.
